# The structure of FIV reverse transcriptase and its implications for non-nucleoside inhibitor resistance

**DOI:** 10.1371/journal.ppat.1006849

**Published:** 2018-01-24

**Authors:** Meytal Galilee, Akram Alian

**Affiliations:** Faculty of Biology, Technion–Israel Institute of Technology, Haifa, Israel; Miller School of Medicine, UNITED STATES

## Abstract

Reverse transcriptase (RT) is the target for the majority of anti-HIV-1 drugs. As with all anti-AIDS treatments, continued success of RT inhibitors is persistently disrupted by the occurrence of resistance mutations. To explore latent resistance mechanisms potentially accessible to therapeutically challenged HIV-1 viruses, we examined RT from the related feline immunodeficiency virus (FIV). FIV closely parallels HIV-1 in its replication and pathogenicity, however, is resistant to all non-nucleoside inhibitors (NNRTI). The intrinsic resistance of FIV RT is particularly interesting since FIV harbors the Y181 and Y188 sensitivity residues absent in both HIV-2 and SIV. Unlike RT from HIV-2 or SIV, previous efforts have failed to make FIV RT susceptible to NNRTIs concluding that the structure or flexibility of the feline enzyme must be profoundly different. We report the first crystal structure of FIV RT and, being the first structure of an RT from a non-primate lentivirus, enrich the structural and species repertoires available for RT. The structure demonstrates that while the NNRTI binding pocket is conserved, minor subtleties at the entryway can render the FIV RT pocket more restricted and unfavorable for effective NNRTI binding. Measuring NNRTI binding affinity to FIV RT shows that the “closed” pocket configuration inhibits NNRTI binding. Mutating the loop residues rimming the entryway of FIV RT pocket allows for NNRTI binding, however, it does not confer sensitivity to these inhibitors. This reveals a further layer of resistance caused by inherent FIV RT variances that could have enhanced the dissociation of bound inhibitors, or, perhaps, modulated protein plasticity to overcome inhibitory effects of bound NNRTIs. The more “closed” conformation of FIV RT pocket can provide a template for the development of innovative drugs that could unlock the constrained pocket, and the resilient mutant version of the enzyme can offer a fresh model for the study of NNRTI-resistance mechanisms overlooked in HIV-1.

## Author summary

The majority of anti-AIDS drugs target the reverse transcriptase (RT) enzyme of the HIV-1 virus. RT catalyzes the central step in the virus replication cycle converting the viral RNA genome into DNA for subsequent integration into the host genome. As with all anti-AIDS treatments, continued success of RT inhibitors is persistently disrupted by the occurrence of resistance mutations. To explore latent resistance mechanisms potentially accessible to therapeutically challenged HIV-1 viruses, we examined RT from the related feline immunodeficiency virus (FIV). FIV closely parallels HIV-1 in its replication and pathogenicity however is resistant to all non-nucleoside inhibitors of HIV-1 RT. We resolved the crystal structure of FIV RT, and using mutational and biochemical analyses, we show that specific differences in the FIV RT structure inhibit the binding of non-nucleoside inhibitors. We further show that mutating the protein to facilitate binding of the inhibitors does not confer sensitivity to these inhibitors, suggesting that other variances inherent in FIV RT modulate a second layer of resistance. These insights can help in the development of novel drugs against evolving HIV-1 RT resistance.

## Introduction

**Fig 1:**
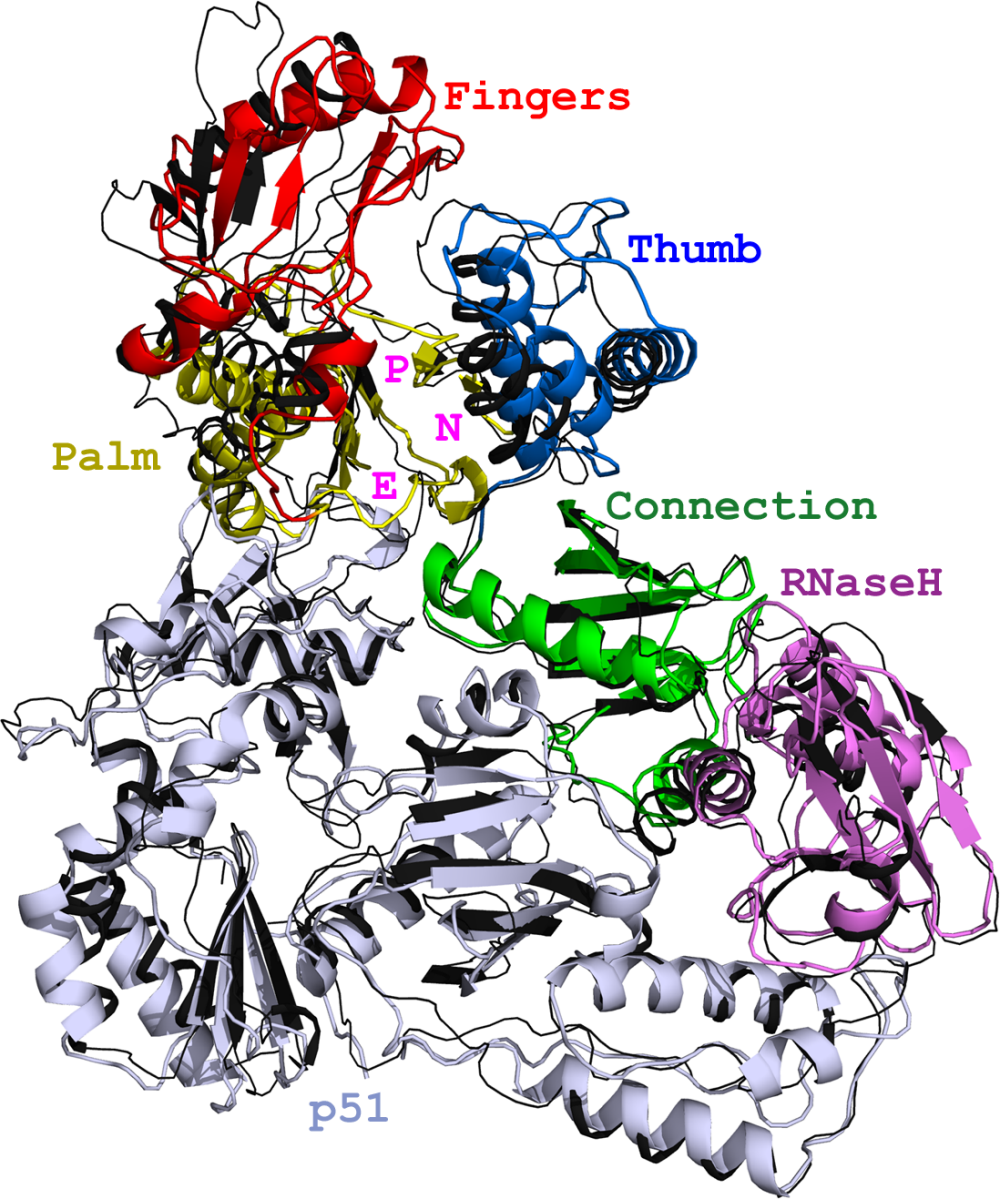
Structure of FIV RT. Superposition of RT from FIV and HIV-1 (black, PDB code: 1DLO). Subdomains of FIV p66 are color coded and designated according to HIV-1 RT [24]. FIV p51 is shown in light blue. Primer grip (P), NNBP (N) and entryway (E) are indicated.

Reverse transcriptase (RT) is the most common target for anti-AIDS drugs being the enzyme that catalyzes the central step in the HIV-1 replication cycle converting the viral RNA genome into DNA for subsequent integration into the host genome [1]. While the relative contribution is still undetermined, errors made by the RT enzyme provide one source of genetic variances emerging in the replicating viral genomes and facilitating the development of resistance to all anti-AIDS drugs [1]. RT inhibitors are mainly nucleoside/nucleotide analogues (NRTI), which target the catalytic site acting as competitive chain terminators in the enzymatic reaction, or non-nucleoside inhibitors (NNRTI) targeting a hydrophobic pocket key in allosteric regulation of RT structural rearrangements [1]. RT is a heterodimeric protein of two subunits, p51 and p66, encoded by the p66 template and, therefore, identical in sequence except for lacking the C-terminal RNase-H domain in p51 as a result of proteolytic processing. The structure of p51 is rigid and provides structural support to the more flexible p66 subunit that undergoes functionally crucial conformational rearrangements. The unliganded p66 predominantly folds into a “closed” conformation of a “right-hand” shape with the “thumb” crumpled down on the “fingers” (Fig 1). Upon nucleic acid binding, the thumb lifts up and fingers fold down to hold an incoming nucleotide for a productive reaction. Within the “palm” subdomain, and adjacent to the flexible thumb, resides a hydrophobic non-nucleoside binding pocket (NNBP) (S5 Fig). By targeting this pocket, NNRTIs restrict the structural flexibility of RT and abolish the DNA polymerization activity of the enzyme [1–3]. Although inhibition mechanisms have yet to be specifically defined, NNRTIs have been suggested to act by restricting the mobility of the thumb, distorting the catalytic triad, repositioning the primer grip and loosening the thumb and fingers clamp (reviewed in [4]).

As with all anti-AIDS treatments, resistance mutations persistently disrupt the continued success of RT inhibitors. While the mechanistic bases are not yet clearly defined, the prevailing view establishes that NNRTI-resistance mutations mainly alter the association and dissociation kinetics of NNRTIs (reviewed in [4]). Resistance mutation K103N has been suggested to restrict the conformational plasticity of NNBP [5], by forming additional hydrogen bonds that stabilize Y188 into a “closed” form of the pocket [6–8], reducing the rate of inhibitor binding [6, 9]. A recent mutational study, however, has shown that while the additional hydrogen bonds may have contributed to resistance (~ 2 fold enhancement), hydrophobic and electrostatic interactions contributed by the K103N played the fundamental role in impacting NNRTI binding kinetics [10]. Similarly, E138K has been shown to enhance the dissociation rate of NNRTIs [11]. Mutations such as E138K, K101E and V179D can alter the chemical environment around the NNBP and its entryway and, therefore, can impact NNRTI association and dissociation rates [4, 5]. E138K and K101E have also been proposed to modulate the conformational plasticity by disrupting the formation of an inter-subdomain salt-bridge, proposed to enhance the inhibitory effect of NNRTIs [5, 9]. Nevertheless, earlier kinetic studies highlight a controversy with this prevailing “binding kinetics” dogma by showing that even at saturating concentrations of NNRTI, a slow but significant polymerization activity continues by the fully inhibited enzyme and not due to the uninhibited enzyme recovered by the slow release of inhibitor [12, 13]. This challenge has recently been emphasized by a study showing that the K103N resistance mutation does not inhibit NNRTI binding to RT, and that NNRTI binding does not prevent the formation of a productive reverse transcription complex [9]. Therefore, we may not completely rule out a potential role of resistance mutations in modulating the conformational plasticity of RT subdomains, enabling the formation of a productive reverse transcription complex of an NNRTI-bound RT.

Current NNRTIs are HIV-1 specific. RT enzymes from HIV-2, simian (SIV) and feline (FIV) immunodeficiency viruses are intrinsically resistant to HIV-1 NNRTIs. The intrinsic resistance of FIV RT (fRT) is particularly interesting since FIV harbors the Y181 and Y188 NNBP sensitivity residues absent in both HIV-2 and SIV (S1 Fig) [14]. Unlike HIV-2 RT, or SIV RT (V181Y/L188Y) [15], where a simple I181Y/L188Y mutation bestowed NNRTI susceptibility [16], previous efforts have failed to make the fRT susceptible to NNRTIs, attributing resistance to distinct structural features potentially crafting a more rigid enzyme [14, 17]. It has been speculated that this rigidity could make the creation of the NNRTI-pocket, and consequent NNRTI penetration or binding, energetically unfavorable, or, alternatively, could adjust subdomain plasticity to mitigate inhibitory impacts of bound NNRTIs [14].

FIV RT inherently combines numerous substitutions frequently observed in HIV-1 resistance to NNRTIs including (HIV-1 numbering used; FIV variances in brackets) M41L, V75M(I), K101E/Q(Q), V118I, I132L, E138K/A(A), Q145M/L(V), V179D, F227L/C(Y), K238T, L283I(M), N348I, I393L(T), T/A376S(R), and T369I/V(L) (S1 Fig) (reviewed in [2, 4, 18], and Stanford Database https://hivdb.stanford.edu/). While the exact role and significance of each of these variances, separately and combined, remains to be determined, fRT presents a pertinent model for probing the NNRTI-resistance mechanisms. The FIV model is the only non-primate model of a lentivirus that induces AIDS-like syndrome in its natural host [19], and has greatly advanced our understanding of fundamental questions in retrovirology [19–21], including drug-resistance in HIV-1 RT and the identification of novel RT inhibitors [22]. The remarkable NNRTI-resistance of FIV RT has stimulated earlier studies exploiting the feline enzyme in probing resistance determinants of RT [14, 17]. The decreased catalytic activity of FIV/HIV-1 chimeric RT of these studies has underscored the significance of the delicately tuned p51/p66 inter-subunit interactions, and pinpointed NNRTI-sensitivity to the p66 subunit [14, 17]. Interestingly, whereas HIV-1 RT has been made entirely insensitive to NNRTIs by exchanging a fragment containing residues 97–205 of FIV RT, the reverse swapping did not bestow NNRTI-susceptibility to FIV RT [14], suggesting a more complex resilience. While the structure and function of FIV proteins are highly conserved with their HIV-1 analogues [20, 21], they greatly diverge in their amino acid sequences. Therefore, exploring how orthologous proteins coevolved in their natural environment can highlight crucially conserved patterns for drug and vaccine targeting, and more importantly, can uncover conceivably latent escape patterns accessible to challenged HIV-1. Here, we report the first crystal structure of RT from a non-primate lentivirus, the FIV, and provide insights into its complex mechanism of NNRTI resistance. The structure reveals a “closed” pocket configuration restricting NNRTI binding potentially by the distinct rigidity and electrostatic features of the pocket. We show that FIV RT is unable to bind NNRTIs and whilst mutating the loop at the NNBP-entryway confers binding ability, it does not provide sensitivity to the inhibitors. Therefore, the feline enzyme appears to harbor a more complex combination of variances that specifically constrain the NNBP pocket attenuating NNRTI binding, and further variances that potentially could enhance the dissociation of bound inhibitors, or, perhaps, modulate protein plasticity to overcome inhibitory effects of bound NNRTIs. Together, the more “closed” conformation of FIV RT pocket providing a unique example of impeded NNRTI-binding, and the resilient mutant version of FIV RT, which offers a model for NNRTI-resistance based on distinct variances inherent in FIV, make the FIV RT an invaluable model for the study of HIV-1 RT resistance and drug development.

## Results and discussion

### The preserved fold of FIV RT

To elucidate the mechanisms employed by fRT and promote our general understanding of NNRTI-resistance, we determined the 2.94 Å crystal structure of fRT (Table 1). With 48% identity and 67% similarity [23] (S1 Fig), the fold of feline RT is conserved with the HIV-1 RT. The p51/p66 heterodimeric enzyme embraces the famous “right-hand” shape of p66 [1, 5, 24], which superimposes on the “closed” form of unliganded HIV-1 RT (~ 2 Å RMSD) (Fig 1).

**Table 1:** Crystallography data collection and refinement statistics.

	FIV RT
**Data collection**	
Space group	P 31 2 1
Mol/ASU Cell dimensions	1
*a*, *b*, *c* (Å)	127.14 127.14 191.34
α, β, γ (°)	90 90 120
Resolution (Å)	60.33–2.94 (3.05–2.94)*
*R*_sym_ or *R*_merge_	0.10 (1.18)
*I* / σ*I*	17.1 (2.2)
Completeness (%)	99.91 (99.76)
Redundancy	9.8 (9.7)
**Refinement**	
Resolution (Å)	2.94
No. unique reflections	37324 (3797)
*R*_work_ / *R*_free_ (5% test set)	0.232 (0.31) / 0.266 (0.36)
No. atoms	
Protein	7431
Water	37
*B*-factors	
Protein	97.1
Water	79.8
R.m.s. deviations	
Bond lengths (Å)	0.006
Bond angles (°) Ramachandran (%) Favored Allowed Outliers PDB code	0.98 92.0 7.7 0.54 5OVN

* Number in parentheses is for highest resolution shell.

### The conserved architecture of the NNRTI-binding pocket

**Fig 2:**
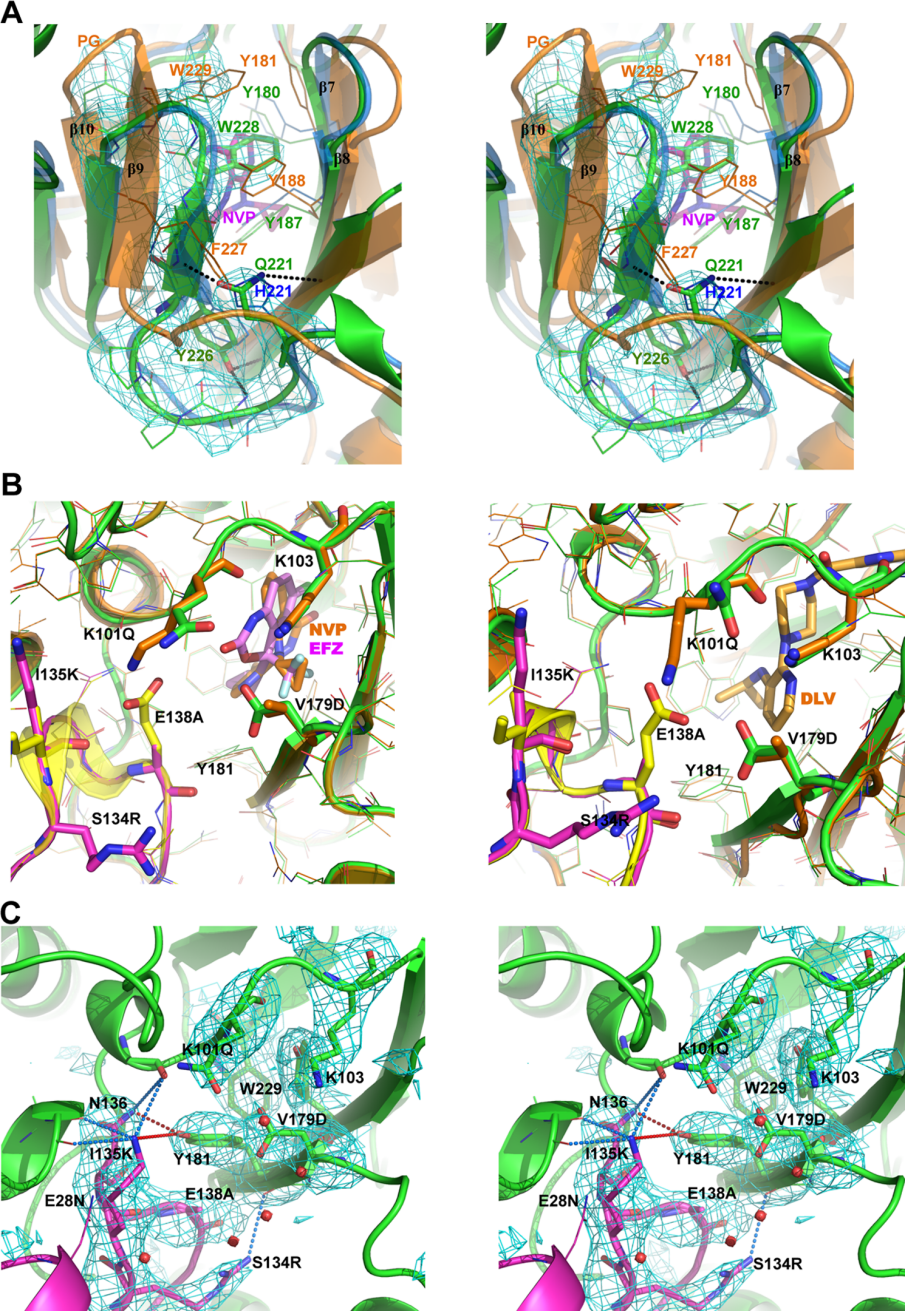
NNRTI-pocket of FIV RT. (**A**) Walleye stereo view depicting the opening of HIV-1 primer-grip (PG) upon NNRTI binding. RT (p66) from FIV (green) is compared to HIV-1 RT (p66) unliganded (blue, PDB code: 1DLO) and NVP (magenta sticks) bound (orange, PDB code: 3HVT). Cyan mesh represents electron density map (F_O_-F_C_, 3.0σ) calculated after omitting fRT residues 222–231. Interactions (< 4 Å) are shown in black dashed lines. (**B**) FIV RT (p66/p51 in green/magenta) was modeled open using HIV-1 RT (p66/p51 in orange/yellow) with PDB codes (left panel) 3HVT for NVP (orange sticks) and 1FK9 for EFZ (magenta sticks), and (right panel) 1KLM for DLV (light-orange sticks). **(C)** Walleye stereo view showing pocket entrance at the interface of fRT p66 (green) and p51 (magenta). Cyan mesh represents electron density map (F_O_-F_C_, 3.0σ) calculated after omitting the labeled residues (excluding E28N) (HIV-1 numbering). Interactions by Y181 and I135K^p51^ are indicated with red and blue dashed lines, respectively (< 4 Å except for Y181-I135K ^p51^ at a distance of 4.5 Å). Red spheres show waters.

The NNBP of unliganded RT is rimmed at the p51/p66 interface (residues 100–105 of p66 and E138-loop of p51) and largely filled by the primer grip (β9-β10) and the catalytic loop (β7-β8) [1, 25]. The pocket opens upon NNRTI binding, which causes the side chains of Y181 and Y188 to flip upwards moving the primer-grip (β9-β10) away from the catalytic β7-β8 loop [1, 4, 5, 24, 26] (Fig 2A). The fRT NNBP structure is conserved with HIV-1 with variances around the NNBP of fRT making no global change to the pocket size or shape. Both FIV and HIV-1 RT NNBPs are of comparable volumes (~635 Å^3^) and superimposable NNRTIs in a model of open fRT NNBP (Fig 2B).

### Minor subtleties at the entryway may lock the NNRTI-pocket

**Fig 3:**
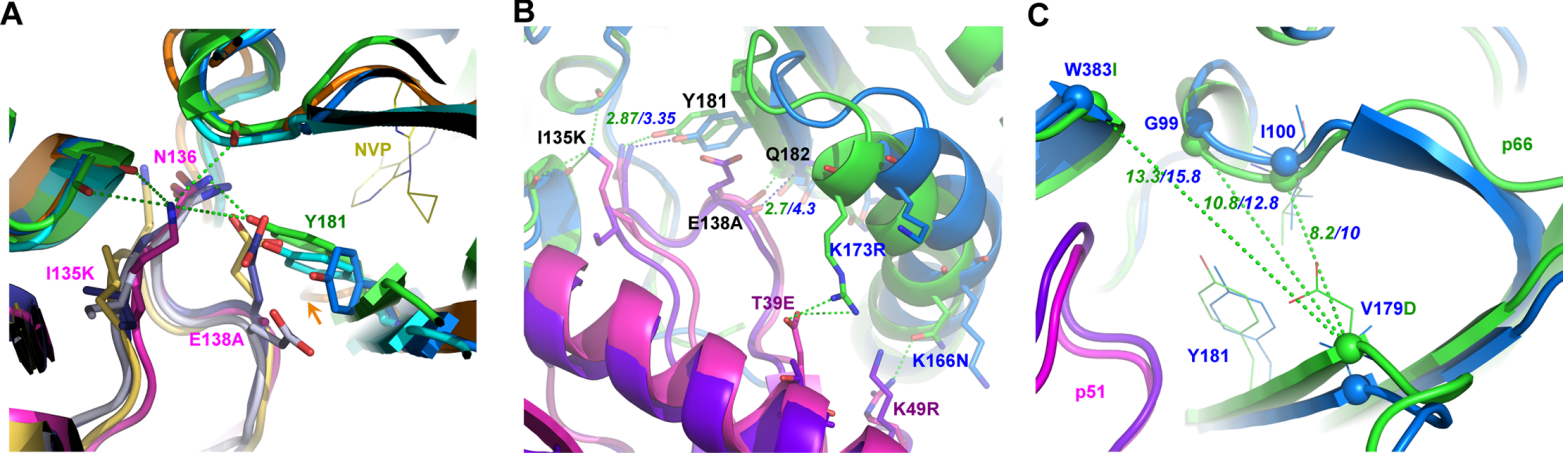
NNRTI-pocket and entryway. (**A**) Pocket entrance of fRT (p66/p51 (green) and p51 (magenta)) and HIV-1 unliganded RT p66/p51 (blue/deep-blue (PDB code: 1HMV) and cyan/blue-white (PDB code: 1DLO)) and NVP-bound (orange/yellow, PDB code: 3HVT). fRT p51/p66 interactions in green dashed lines. Arrow (orange) indicates open position of Y181 (orange) in NVP-bound structure (orange/yellow, PDB code: 3HVT). (**B**) fRT forms additional bonds between p51 (magenta (FIV) and purple (HIV-1)) and p66 (green (FIV) and blue (HIV-1)) around the NNRTI-pocket entrance: one salt-bridge (K173R/T39E ^p51^, 2.98 Å), and two hydrogen bonds (K166N/K49R ^p51^ at 2.89 Å, and backbone amine of Q182 with carbonyl oxygen of E138A ^p51^ at 2.7 Å in fRT but 4.3 Å in HIV-1 RT (PDB code: 1DLO)). Distances (green dashed lines, Å) are shown for fRT (green numbers) and HIV-1 RT (blue numbers). (**C**) Comparison of pocket entrance of RT p66 from FIV (green) and HIV-1 (blue, PDB code: 1DLO). Spheres indicate Cα of the labeled residues (FIV substitutions in green font). Distances (green dashed lines) of HIV-1 (blue font) are average of 6 structures of unliganded RT (PDB codes: 1DLO, 1HMV, 1QE1, 1HQE, 3DLK and 3KLI) and indicate distances (Å) between Cα of V179 and of I100 (10 ± 0.33 Å), G99 (12.8 ± 0.43 Å) or W383 (15.8 ± 0.46 Å). Averaging the three distance-variances show that FIV entryway is smaller by 2.1 Å. p51 of HIV-1 (purple) and FIV (magenta) are shown.

Although the NNBP of FIV and HIV-1 RT are conserved, several neighboring variances could modulate the chemical features and plasticity of the rims walling the NNBP and its entrance, perhaps making it unfavorable for NNRTIs to penetrate and bind. Because p51 is a proteolytic product of p66, variances or mutations will be identical in both subunits. However, we refer to variances specific to each subunit where structurally relevant (e.g. at the p51/p66 interface). Intriguingly, a group of FIV variances clusters at the rim of the NNBP entryway. fRT is altered to K101Q, D192N, D177Q and V179D in the p66 subunit, and E28N, T139G, E138A, I135K and S134R, in the p51. The prominent contributors to key physiochemical alterations at the entryway appear to be K101Q and V179D of p66, and E138A^p51^ and I135K^p51^ of p51 (Figs 2C and 3A).

Structural analysis suggests that variances at the fRT entryway could render a rigid pocket with a more of a “closed” conformation that could inhibit NNRTI binding. Particularly, Y181, which must flip upwards to open the NNBP, appears to be trapped by interactions with the conserved N136^p51^ and potentially with I135K^p51^. In HIV-1 RT, the negatively charged and bulky side chain of E138^p51^ may shield Y181 against these interactions (Figs 2C and 3A). In an unliganded HIV-1 RT structure (PDB code: 1HMV) E138^p51^ is positioned between Y181 and N136^p51^ (spaced at 6.7 Å), preventing their interaction. In another HIV-1 RT structure (PDB code: 1DLO) E138^p51^ is rotated away from Y181, allowing a 3.4 Å interaction between Y181 and N136^p51^, 0.5 Å farther, and consequently weaker, than the interaction in fRT (2.9 Å) (Fig 3A and 3B). Moreover, I135K^p51^ stabilizes the p51/p66 interface with potential interactions (< 4 Å) to carbonyl oxygen of three p66 residues (98, 382 and 383). The bulkier side chain of S134R^p51^ may further contribute to a tighter entryway by filling more space at the p51/p66 interface and interacting (3.6 Å) with the carbonyl oxygen of I180 (Fig 2). fRT also forms three additional bonds around the entryway: one salt bridge (K173R/T39E^p51^) and two hydrogen bonds (K166N/K49R^p51^, and backbone amine of Q182 with carbonyl oxygen of E138A^p51^) (Fig 3B). Consequently, the pocket entrance of FIV p66 is constricted by ~ 2 Å as compared to that of HIV-1 (Fig 3C). FIV variances, especially at the p51 loop (S134R, I135K, E138A and T139G), have also altered the electrostatic properties around the entryway from negative to positive charges, which may interfere with initial drug channeling into the entrance and subsequent penetration and binding (S2 Fig).

Additionally, a cluster of substitutions in fRT creates extra interactions that could restrain the flexibility of the primer grip (β9-β10) rimming the NNBP. Particularly, Gln of H221Q in fRT forms an extra interaction to backbone amine of L228T. Also, OH of F227Y interacts with backbone amines of V106 or K223E (Fig 2A). These interactions may further stabilize the fRT primer-grip, making its displacement and Y188/W229 dislocation out of the NNBP energetically unfavorable. Collectively, FIV variances at the p51/p66 interface appear to have promoted a more rigid and “closed” pocket conformation impeding NNRTI penetration and binding. Whether the “locked” configuration is a consequence of the extra interactions making the pocket more rigid, or a consequence of the electrostatic features at the entryway, needs to be validated by future studies.

### The locked pocket configuration inhibits NNRTI binding

**Fig 4:**
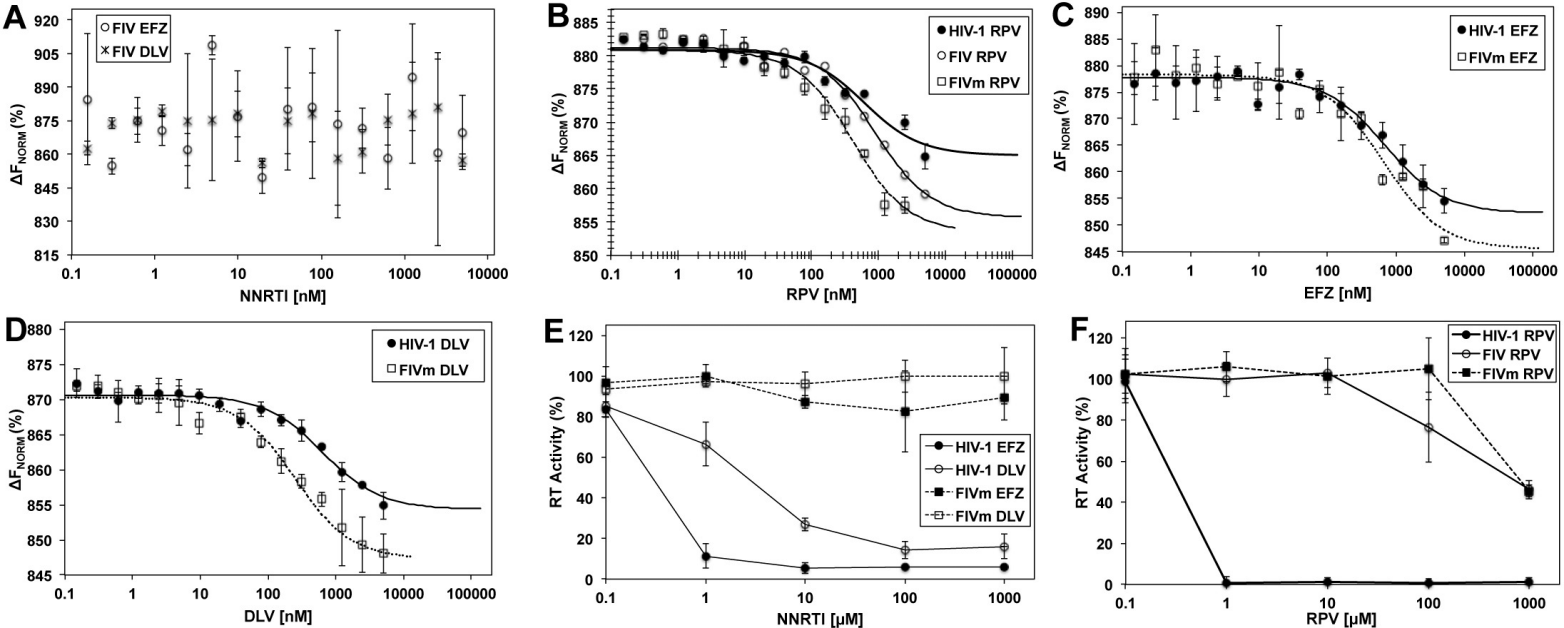
NNRTIs binding and RT activity. **(A)** EFZ and DLV binding to fRT. **(B)** RPV binding to fRT (Kd 796 ± 76 nM) and mutant fRT (FIVm) (Kd 368 ± 60 nM) as compared to HIV-1 RT (Kd 539 ± 25 nM). P = 0.01 (one-way ANOVA) for FIVm when compared to fRT (FIV), but non-significant (0.11) when compared to HIV-1. **(C)** EFZ binding to mutant fRT (FIVm) (Kd 634 ± 88 nM) as compared to HIV-1 RT (Kd 675 ± 122 nM). **(D)** DLV binding to mutant fRT (FIVm) (Kd 227 ± 71 nM) as compared to HIV-1 RT (Kd 617 ± 100.4). P < 0.05 (one-way ANOVA) for FIVm when compared to HIV-1. Values are means of duplicates from two (three in RPV) biological repeats (±SD). ΔF_Norm_: normalized fluorescence units (% of bound and unbound substrate). **(E)** DNA polymerization activity of RT from HIV-1 (black lines) and FIV mutant (FIVm, dashed lines) as affected by EFZ (black marks), DLV (clear marks) or **(F)** RPV. Values are means of triplicates from two biological repeats (six repeats for RPV) (±SD). Values are band-density quantifications of gels resolving the polymerized DNA product (S4B Fig).

The closed architecture of the NNBP entryway readily indicates that NNRTI binding to fRT would not be favorable. Previous studies documented the failure of NNRTIs to inhibit fRT activity but did not establish if this is a result of fRT inability to bind NNRTIs [14, 17]. In order to clarify this point, we measured the binding affinities of fRT to Delavirdine (DLV), Efavirenz (EFZ) and Rilpivirine (RPV). DLV is a first generation NNRTI inhibitor, susceptible to resistance, whereas EFZ and RPV are potent second generation inhibitors [1]. We found that DLV and EFZ inhibitors fail to bind to fRT (Fig 4A), supporting our model of a restricted NNRTI-pocket in the feline enzyme. Surprisingly, the more flexible RPV inhibitor, which has not previously been assessed with feline enzyme, was able to bind to fRT with comparable affinity to HIV-1 RT (Fig 4B), providing a promising molecule that could overcome the physiochemical barriers imposed by fRT variances at the pocket entryway.

To further validate the restricted-pocket configuration, we mutated the fRT p51-loop to match its HIV-1 RT equivalent (R133S, K134I, A137E, G138T and R141I in FIV p51) and found that the mutated fRT was able to bind EFZ and RVP with comparable affinities to HIV-1 RT (Fig 4B & 4C) and binds DLV with twice the affinity as compared to HIV-1 RT (Fig 4D). RPV bound to the mutant with twice the affinity as compared to fRT (Fig 4B). These results support our proposed “closed” pocket configuration of the feline enzyme, which was opened by mutating residues gating the pocket entryway.

Noteworthy, the fRT mutant had reduced thermal stability (~ 2.5°C), presumably as a result of the interactions lost through this mutation (S3 Fig).

### FIV RT mutant maintains resistance to NNRTIs

The structure of the intrinsically resistant fRT provides a novel model of a constrained NNRTI pocket, which when combined with the numerous other NNRTI-resistance mutations inherent in fRT could result in a resilient enzyme. Although mutating the p51-loop opened the NNBP for drug binding, this was not sufficient to render fRT sensitive to these drugs. While potently inhibiting HIV-1 RT, none of the three inhibitors EFZ, DLV or RPV reduced the activity of the mutated fRT: the mutant retained the same activity as wild type fRT in both the presence and absence of these NNRTIs (Fig 4E, 4F and S4 Fig). Although RPV was able to bind the wild type fRT enzyme (Fig 4B), it was unable to inhibit its polymerization activity (Fig 4E), further supporting our observation that the fRT mutant, rendered susceptible to NNRTI binding, continued to resist inhibition. Whereas both K101Q and V179D variances inherent in fRT, similar to HIV-1 NNRTI-resistance mutations (see introduction), could modulate the chemical environment gating the pocket entryway (Fig 2B) enhancing inhibitor dissociation, a Q101K/D179V mutation of fRT was not sufficient to render it susceptible to NNRTIs [14], and therefore may not alone, explain fRT resistance to RPV that we observed (Fig 4F). A comprehensive mutational analysis coupled with detailed kinetic study would be required to establish the mechanistic basis underlying the continue resistance of the fRT, which binds RPV, and the fRT mutant, which binds all three NNRTIs. Whereas the pocket-opening mutation promoted inhibitor binding, analysis of dissociation kinetics is required to establish the role of the many other fRT variances in enhancing NNRTI dissociation or, perhaps, accentuating an overlooked role of these variances in facilitating a catalytically competent form of an inhibitor-bound enzyme, which has previously been documented [9, 12, 13]. The fRT and its p51-mutant offer pertinent models for such kinetic and mechanistic studies that could advance our understanding of the mechanisms underlying NNRTI resistance of lentiviral RT.

### Potential significance of variances near the nucleotide-site

Variances distinct to fRT clustering around the nucleotide-binding site may also add to the overall resilience of the feline enzyme. A cross-species study investigating FIV virus evolution revealed that the majority of positive-selection mutations mainly cluster in the nucleotide-binding pocket and sites contacting nucleic acid substrates [27]. Modeling fRT bound to a nucleotide shows that, additional to more common variances (I47V, K73M, V75I, V111I and L214F), FIV contains unique substitutions, e.g. L12M, M41L, D67-lacking, F124Y, Y146W, V148S, K154I, G155L and F160Y, and three potential salt-bridges to form by T215E (to K46), K219D (to K70) and K223E (to I195K and R199K) (S1 Fig and S5 Fig). For example, V111I mutation in HIV-2 has been proposed to constrain the mobility of a flexible loop resulting in a productive increase in nucleotide pocket accessibility [28]. Therefore, while the exact role of these variances remain to be determined, their contribution to NNRTI-resistance should not be ignored and is reminiscent to NNRTI-resistance mutations of HIV-1.

Given its importance for productive virus infectivity, it is not surprising to find that RT accumulates the majority of positive-selection mutations during a cross-species study investigating virus evolution [27]. NNBP residues are not essentially conserved, therefore, HIV-1 has a rather low genetic barrier for developing NNRTI-resistance mutations [1]. The 2016 Los Alamos database (4416 entries) (https://www.hiv.lanl.gov/) shows that distinct substitutions of fRT p51 can occur in HIV-1 p51: K101Q (0.32%, K101R: 0.45%), I135K (0.38%, I135R: 1.54%), E138A (1.88%), and S134R (3 occurrences combined with E138A or S134N). Of the 14 records of K101Q, one was combined with E138A and 4 with I135R. There was yet no record of I135K/R with E138A. The resistant mutant version of FIV RT, combining these mutations together, may now provide a model to predict novel resistance mechanisms that a challenged HIV-1 may exploit in escaping inhibition of bound NNRTIs. The resilient FIV mutant RT offers a model for NNRTI-resistance based on distinct variances inherent in FIV but overlooked in HIV-1, HIV-2 and SIV. These intrinsic species-specific differences, which apparently hinge on variations as subtle as few or single residue mutations, highlight the importance of cross-species analysis for discovering potential unknown alternative routes awaiting HIV-1 exploitation. However, while NNBP residues are not crucially conserved and recombinant RT mutants were found enzymatically active, future studies need to evaluate the replication competence of FIV and HIV-1 viruses containing the p51-loop mutations.

Prominently, the wild type FIV RT offers a unique model to a more constrained pocket than has previously been observed in other lentiviruses and may, therefore, pave the way for the development of novel NNRTIs that need to disrupt such a locked pocket that may emerge in HIV-1. Whereas RPV appears to be one such inhibitor, further studies need to investigate whether the observed RPV binding to wild type fRT (Fig 4B) is specific to NNBP and what makes the fRT enzyme RPV resistant (Fig 4F). This study opens new horizons and opportunities to elaborate on minimal p51 variances required to block NNBP entryway, as well as detailed binding kinetics of present and novel NNRTIs to further our understanding of NNRTI-resistance mechanisms.

## Materials and methods

### Protein expression, purification and crystallization

The two subunits of FIV RT (Petaluma) were subcloned into separate plasmids to facilitate site-specific mutagenesis. The p66 subunit (residues 1–554) was cloned into pET22b (*amp* resistance, Novagen) and p51 subunit (residues 1–430) in pET28b (*kan* resistance, Novagen) with a thrombin-cleavable His-tag at the N-terminus. FIV RT mutant (FIVm) was generated by introducing the specific mutations into the p51 subunit using QuikChange Kit (Agilent Technologies Genomics). HIV-1 RT construct, containing p66 and protease coding regions in pT5M plasmid, was a generous gift of Prof. Stephen H. Hughes (National Cancer Institute- Frederick, MD, USA).

FIV RT constructs were expressed in ArcticExpress cells (Agilent Technologies Genomics) with 0.5mM IPTG induction overnight in 16°C. HIV-1 RT was expressed in BL21(DE3) with 1mM IPTG induction for 4 hours in 37°C.

RT proteins from FIV and HIV-1 were purified using HisPur Cobalt Resin (Thermo Fisher Scientific) in a buffer containing 500 mM NaCl, 20mM potassium phosphate pH 7.5, 5 mM β-mercaptoethanol and dialyzed into a final buffer containing 500mM NaCl, 20mM Hepes 6.0, 2 mM DTT. Thrombin (2 units/mg, Novagen) was used to cleave the His-tag of FIV RT (4°C, overnight), and was subsequently removed using benzamidine sepharose resin (GE Healthcare Life Sciences). FIV RT proteins were further purified using HiTrap SP HP cation exchange (GE Healthcare Life Sciences) eluting at 350 mM NaCl.

For crystallization, FIV RT was further purified using Superdex-200 size exclusion column (GE Healthcare Life Sciences) equilibrated with 300 mM NaCl, 20mM Hepes pH 7.4 and 2mM DTT, and concentrated to 10 mg/ml. Protein crystals were grown using the hanging-drop vapor-diffusion method at 4°C. Initial crystals were obtained in C9 condition of crystals screen (Hampton Research) containing 4.0 M sodium formate. Diffraction quality crystals were obtained in 3.8 M sodium formate supplemented with 0.2 M ammonium formate. Cryoprotectant solutions contained an additional 20% glycerol.

### Structure determination

Diffraction data were collected at beamline I-24 of Diamond Light Source Ltd (Harwell Research and Innovation Campus, UK), and processed with XDSAPP [29]. The structure was solved by molecular replacement using BALBES [30] and 3DOL [31] as a search model. The resulting model was fitted into the electron densities using COOT [32] and refined using REFMAC5 [33] in CCP4 suite [34]. Table 1 summarizes data collection and refinement statistics.

### Activity assay

DNA-dependent DNA polymerization activity of RT enzymes was assessed using a DNA primer/template prepared by annealing of 70-mer ssDNA template with a complementary 25-mer ssDNA primer labeled with 6-FAM at 5’-end (Integrated DNA Technologies).

Reaction mixtures contained 100 mM Tris-HCl pH 7.5, 75 mM KCl, 0.5 mM dNTP mixture, 60 mM MgCl2, 5 mM DTT and 0.15 μM RT, 0.5 μM DNA substrate. Reactions were incubated for 30 minutes at 37°C and stopped using urea sample loading buffer and heating at 95°C for 10 minutes. Reaction products were resolved using denaturing urea polyacrylamide gel electrophoresis (S5B Fig). Percentage RT activities represent percentile of band-intensities of polymerized product divided by those of template/primer substrate.

### Affinity measurements

RT proteins were labeled with Monolith NT protein labeling kit BLUE-NHS (NanoTemper Technologies) and eluted in microscale thermophoresis (MST) buffer (50 mm Tris-HCl, pH 7.0, 300 mM NaCl and 0.05% Tween-20). Labeled RT proteins were incubated with serial dilutions of Efavirenz or Delaviridine, and MST measurements were carried out using the Monolith NT.115 in standard capillaries with 40% LED and 20% MST power.

### NanoDSF measurements

RT proteins were diluted (into 20 mM Hepes pH 7.4 and 300 mM NaCl) to reach a final concentration of 0.15 mg/ml and subsequently filled into standard treated capillaries. Label-free differential scanning fluorimetry (nanoDSF) measurements were carried out using Prometheus NT.48 (NanoTemper Technologies) and thermal unfolding was monitored with temperature changing from 15°C to 95°C, with the rate of 1°C/min.

### Sequence and structural analysis

Protein sequences were aligned using T-Coffee [35] and annotated using ESPript [36]. PyMOL Molecular Graphics System (Schrödinger, LLC) was used for preparing structural figures and for structural superposition. Comparative pocket volumes were analyzed using the CASTp server [37] using HIV-1 RT bound to NVP without nucleic acid (PDB code: 5HBM) or bound to NVP with nucleic acid (PDB code: 4PUO). Both HIV-1 RT structures show comparable volumes validating the use of this measure. Next, the fRT was modeled into an open conformation using homology modeling and employing these two HIV-1 RT structures as templates. Pocket volumes of fRT and HIV-1 RT were, therefore, compared using one specific NVP inhibitor, and the analysis is only comparative and is not made to indicate an exact volume of NNRTI pockets. Homology modeling was performed using the Swiss Model server [38]. Analyses of interacting interfaces were performed using PDBsum [39].

## Supporting information

S1 Fig Alignment of RT sequences. Sequences of RT from HIV-1, HIV-2, SIV and FIV are compared. Top numbering and secondary structure are shown for HIV-1 (PDB code: 5HBM) and are shown for FIV RT at the bottom. Identical residues are shown in bold red font and conserved equivalents in red font. NNRTI-resistance mutations known in HIV-1 and intrinsic in FIV are in blue. FIV substitutions in known HIV-1 NNRTI-resistance positions are in pink except for two well-investigated mutations K101 and E138 in cyan.
(TIF)

S2 Fig NNRTI-pocket entryway. Electrostatic surfaces of RT from FIV (left) and HIV-1 (right, PDB code: 1DLO) are displayed over cartoon representation. Indicated are Y181 (red asterisk) and p51 loop (black asterisk), which is positively charged (blue) in fRT and negatively charged (red) in HIV-1 RT.
(TIF)

S3 Fig Thermal stability of FIV RT. A ratiometric measurement of the fluorescent signal (Ratio) is plotted against increasing temperature for wild type FIV RT (FIV, black line) and the p51 mutant (FIVm, gray line). The melting temperatures (T_m_) of wild type RT (45.8 ± 0.7°C) and mutant (43.1 ± 0.1°C) are mean values (with standard deviations) of two repeats (each with triplicate measures) and are presented with black and gray vertical lines, respectively. The difference in T_m_ (2.7°C) is statistically significant (P-value = 0.0004, one-way ANOVA). For clarity, the Y-axis scale of FIVm is shifted downwards by 0.03 ratio points.
(TIF)

S4 Fig NNRTIs effect on RT activity. **(A)** DNA polymerization activity of wild type FIV RT as affected by EFZ (black marks) and DLV (clear marks). **(B)** Representative gels used in the quantification of NNRTI effect on activity. Gels show the resolution of the fluorescent-primer/template substrate (PT) and the polymerization product (DNA), which is fluorescently labeled only if the fluorescent-primer was elongated by RT activity.
(TIF)

S5 Fig Model of FIV RT bound to DNA and dNTP. Walleye stereo view of FIV RT (green cartoon) after being modeled bound to DNA (orange) and dNTP (magenta sticks) using HIV-1 RT complex structure (dark yellow, PDB code: 3V4I). EFZ (red stick-spheres) and dislocated primer-grip (β9/10) and catalytic-loop (β7/8) (purple cartoon) are modeled using was modeled using 1FK9. Potential salt bridges are indicated with black dashed lines.
(TIF)

## References

[1] Das K, Arnold E. HIV-1 reverse transcriptase and antiviral drug resistance. Part 1. Curr Opin Virol. 2013;3(2):111–8. doi: 10.1016/j.coviro.2013.03.012 ; PubMed Central PMCID: PMCPMC4097814. 23602471 10.1016/j.coviro.2013.03.012 PMC4097814

[2] Menendez-Arias L, Betancor G, Matamoros T. HIV-1 reverse transcriptase connection subdomain mutations involved in resistance to approved non-nucleoside inhibitors. Antiviral Res. 2011;92(2):139–49. doi: 10.1016/j.antiviral.2011.08.020 . 21896288 10.1016/j.antiviral.2011.08.020

[3] Sarafianos SG, Marchand B, Das K, Himmel DM, Parniak MA, Hughes SH, et al Structure and function of HIV-1 reverse transcriptase: molecular mechanisms of polymerization and inhibition. J Mol Biol. 2009;385(3):693–713. doi: 10.1016/j.jmb.2008.10.071 ; PubMed Central PMCID: PMCPMC2881421. 19022262 10.1016/j.jmb.2008.10.071 PMC2881421

[4] Das K, Arnold E. HIV-1 reverse transcriptase and antiviral drug resistance. Part 2. Curr Opin Virol. 2013;3(2):119–28. doi: 10.1016/j.coviro.2013.03.014 ; PubMed Central PMCID: PMCPMC4097817. 23602470 10.1016/j.coviro.2013.03.014 PMC4097817

[5] Sharaf NG, Ishima R, Gronenborn AM. Conformational Plasticity of the NNRTI-Binding Pocket in HIV-1 Reverse Transcriptase: A Fluorine Nuclear Magnetic Resonance Study. Biochemistry. 2016;55(28):3864–73. doi: 10.1021/acs.biochem.6b00113 ; PubMed Central PMCID: PMCPMC4955860. 27163463 10.1021/acs.biochem.6b00113 PMC4955860

[6] Hsiou Y, Ding J, Das K, Clark AD Jr., Boyer PL, Lewi P, et al The Lys103Asn mutation of HIV-1 RT: a novel mechanism of drug resistance. J Mol Biol. 2001;309(2):437–45. doi: 10.1006/jmbi.2001.4648 . 11371163 10.1006/jmbi.2001.4648

[7] Ren J, Milton J, Weaver KL, Short SA, Stuart DI, Stammers DK. Structural basis for the resilience of efavirenz (DMP-266) to drug resistance mutations in HIV-1 reverse transcriptase. Structure. 2000;8(10):1089–94. . 11080630 10.1016/s0969-2126(00)00513-x

[8] Rodriguez-Barrios F, Gago F. Understanding the basis of resistance in the irksome Lys103Asn HIV-1 reverse transcriptase mutant through targeted molecular dynamics simulations. J Am Chem Soc. 2004;126(47):15386–7. doi: 10.1021/ja045409t . 15563158 10.1021/ja045409t

[9] Schauer GD, Huber KD, Leuba SH, Sluis-Cremer N. Mechanism of allosteric inhibition of HIV-1 reverse transcriptase revealed by single-molecule and ensemble fluorescence. Nucleic Acids Res. 2014;42(18):11687–96. doi: 10.1093/nar/gku819 ; PubMed Central PMCID: PMCPMC4191400. 25232099 10.1093/nar/gku819 PMC4191400

[10] Lai MT, Munshi V, Lu M, Feng M, Hrin-Solt R, McKenna PM, et al Mechanistic Study of Common Non-Nucleoside Reverse Transcriptase Inhibitor-Resistant Mutations with K103N and Y181C Substitutions. Viruses. 2016;8(10). doi: 10.3390/v8100263 ; PubMed Central PMCID: PMCPMC5086599. 27669286 10.3390/v8100263 PMC5086599

[11] Singh K, Marchand B, Rai DK, Sharma B, Michailidis E, Ryan EM, et al Biochemical mechanism of HIV-1 resistance to rilpivirine. J Biol Chem. 2012;287(45):38110–23. doi: 10.1074/jbc.M112.398180 ; PubMed Central PMCID: PMCPMC3488081. 22955279 10.1074/jbc.M112.398180 PMC3488081

[12] Spence RA, Kati WM, Anderson KS, Johnson KA. Mechanism of inhibition of HIV-1 reverse transcriptase by nonnucleoside inhibitors. Science. 1995;267(5200):988–93. . 7532321 10.1126/science.7532321 PMC7526747

[13] Spence RA, Anderson KS, Johnson KA. HIV-1 reverse transcriptase resistance to nonnucleoside inhibitors. Biochemistry. 1996;35(3):1054–63. doi: 10.1021/bi952058+ . 8547241 10.1021/bi952058+

[14] Auwerx J, Esnouf R, De Clercq E, Balzarini J. Susceptibility of feline immunodeficiency virus/human immunodeficiency virus type 1 reverse transcriptase chimeras to non-nucleoside RT inhibitors. Mol Pharmacol. 2004;65(1):244–51. doi: 10.1124/mol.65.1.244 . 14722257 10.1124/mol.65.1.244

[15] Ambrose Z, Boltz V, Palmer S, Coffin JM, Hughes SH, Kewalramani VN. In vitro characterization of a simian immunodeficiency virus-human immunodeficiency virus (HIV) chimera expressing HIV type 1 reverse transcriptase to study antiviral resistance in pigtail macaques. J Virol. 2004;78(24):13553–61. doi: 10.1128/JVI.78.24.13553-13561.2004 ; PubMed Central PMCID: PMCPMC533891. 15564466 10.1128/JVI.78.24.13553-13561.2004 PMC533891

[16] Shih CK, Rose JM, Hansen GL, Wu JC, Bacolla A, Griffin JA. Chimeric human immunodeficiency virus type 1/type 2 reverse transcriptases display reversed sensitivity to nonnucleoside analog inhibitors. Proc Natl Acad Sci U S A. 1991;88(21):9878–82. ; PubMed Central PMCID: PMCPMC52824. 1719542 10.1073/pnas.88.21.9878 PMC52824

[17] Auwerx J, North TW, Preston BD, Klarmann GJ, De Clercq E, Balzarini J. Chimeric human immunodeficiency virus type 1 and feline immunodeficiency virus reverse transcriptases: role of the subunits in resistance/sensitivity to non-nucleoside reverse transcriptase inhibitors. Mol Pharmacol. 2002;61(2):400–6. . 11809865 10.1124/mol.61.2.400

[18] Hachiya A, Kodama EN, Sarafianos SG, Schuckmann MM, Sakagami Y, Matsuoka M, et al Amino acid mutation N348I in the connection subdomain of human immunodeficiency virus type 1 reverse transcriptase confers multiclass resistance to nucleoside and nonnucleoside reverse transcriptase inhibitors. J Virol. 2008;82(7):3261–70. doi: 10.1128/JVI.01154-07 ; PubMed Central PMCID: PMCPMC2268505. 18216099 10.1128/JVI.01154-07 PMC2268505

[19] Bienzle D. FIV in cats—a useful model of HIV in people? Vet Immunol Immunopathol. 2014;159(3–4):171–9. doi: 10.1016/j.vetimm.2014.02.014 . 24636302 10.1016/j.vetimm.2014.02.014

[20] Khwaja A, Galilee M, Marx A, Alian A. Structure of FIV capsid C-terminal domain demonstrates lentiviral evasion of genetic fragility by coevolved substitutions. Sci Rep. 2016;6:24957 doi: 10.1038/srep24957 ; PubMed Central PMCID: PMCPMC4840305. 27102180 10.1038/srep24957 PMC4840305

[21] Galilee M, Alian A. Identification of Phe187 as a crucial dimerization determinant facilitates crystallization of a monomeric retroviral integrase core domain. Structure. 2014;22(10):1512–9. doi: 10.1016/j.str.2014.08.001 . 25199694 10.1016/j.str.2014.08.001

[22] Hizi A, Herschhorn A. Retroviral reverse transcriptases (other than those of HIV-1 and murine leukemia virus): a comparison of their molecular and biochemical properties. Virus Res. 2008;134(1–2):203–20. doi: 10.1016/j.virusres.2007.12.008 . 18291546 10.1016/j.virusres.2007.12.008

[23] Amacker M, Hottiger M, Hubscher U. Feline immunodeficiency virus reverse transcriptase: expression, functional characterization, and reconstitution of the 66- and 51-kilodalton subunits. J Virol. 1995;69(10):6273–9. ; PubMed Central PMCID: PMCPMC189525. 7545246 10.1128/jvi.69.10.6273-6279.1995 PMC189525

[24] Kohlstaedt LA, Wang J, Friedman JM, Rice PA, Steitz TA. Crystal structure at 3.5 A resolution of HIV-1 reverse transcriptase complexed with an inhibitor. Science. 1992;256(5065):1783–90. . 1377403 10.1126/science.1377403

[25] Das K, Martinez SE, Bauman JD, Arnold E. HIV-1 reverse transcriptase complex with DNA and nevirapine reveals non-nucleoside inhibition mechanism. Nat Struct Mol Biol. 2012;19(2):253–9. doi: 10.1038/nsmb.2223 ; PubMed Central PMCID: PMCPMC3359132. 22266819 10.1038/nsmb.2223 PMC3359132

[26] Thammaporn R, Yagi-Utsumi M, Yamaguchi T, Boonsri P, Saparpakorn P, Choowongkomon K, et al NMR characterization of HIV-1 reverse transcriptase binding to various non-nucleoside reverse transcriptase inhibitors with different activities. Sci Rep. 2015;5:15806 doi: 10.1038/srep15806 ; PubMed Central PMCID: PMCPMC4625163. 26510386 10.1038/srep15806 PMC4625163

[27] Poss M, Ross HA, Painter SL, Holley DC, Terwee JA, Vandewoude S, et al Feline lentivirus evolution in cross-species infection reveals extensive G-to-A mutation and selection on key residues in the viral polymerase. J Virol. 2006;80(6):2728–37. doi: 10.1128/JVI.80.6.2728-2737.2006 ; PubMed Central PMCID: PMCPMC1395431. 16501082 10.1128/JVI.80.6.2728-2737.2006 PMC1395431

[28] Deuzing IP, Charpentier C, Wright DW, Matheron S, Paton J, Frentz D, et al Mutation V111I in HIV-2 reverse transcriptase increases the fitness of the nucleoside analogue-resistant K65R and Q151M viruses. J Virol. 2015;89(1):833–43. doi: 10.1128/JVI.02259-14 ; PubMed Central PMCID: PMCPMC4301157. 25355888 10.1128/JVI.02259-14 PMC4301157

[29] Krug M, Weiss MS, Heinemann U, Mueller U. XDSAPP: a graphical user interface for the convenient processing of diffraction data using XDS. Journal of Applied Crystallography. 2012;45(3):568–72. doi: 10.1107/S0021889812011715

[30] Long F, Vagin AA, Young P, Murshudov GN. BALBES: a molecular-replacement pipeline. Acta crystallographica Section D, Biological crystallography. 2008;64(Pt 1):125–32. Epub 2007/12/21. doi: 10.1107/S0907444907050172 ; PubMed Central PMCID: PMC2394813. 18094476 10.1107/S0907444907050172 PMC2394813

[31] Ren J, Chamberlain PP, Stamp A, Short SA, Weaver KL, Romines KR, et al Structural basis for the improved drug resistance profile of new generation benzophenone non-nucleoside HIV-1 reverse transcriptase inhibitors. J Med Chem. 2008;51(16):5000–8. doi: 10.1021/jm8004493 . 18665583 10.1021/jm8004493

[32] Emsley P, Lohkamp B, Scott WG, Cowtan K. Features and development of Coot. Acta crystallographica Section D, Biological crystallography. 2010;66(Pt 4):486–501. Epub 2010/04/13. doi: 10.1107/S0907444910007493 ; PubMed Central PMCID: PMC2852313. 20383002 10.1107/S0907444910007493 PMC2852313

[33] Murshudov GN, Skubak P, Lebedev AA, Pannu NS, Steiner RA, Nicholls RA, et al REFMAC5 for the refinement of macromolecular crystal structures. Acta crystallographica Section D, Biological crystallography. 2011;67(Pt 4):355–67. Epub 2011/04/05. doi: 10.1107/S0907444911001314 ; PubMed Central PMCID: PMC3069751. 21460454 10.1107/S0907444911001314 PMC3069751

[34] Winn MD, Ballard CC, Cowtan KD, Dodson EJ, Emsley P, Evans PR, et al Overview of the CCP4 suite and current developments. Acta crystallographica Section D, Biological crystallography. 2011;67(Pt 4):235–42. Epub 2011/04/05. doi: 10.1107/S0907444910045749 ; PubMed Central PMCID: PMC3069738. 21460441 10.1107/S0907444910045749 PMC3069738

[35] Notredame C, Higgins DG, Heringa J. T-Coffee: A novel method for fast and accurate multiple sequence alignment. J Mol Biol. 2000;302(1):205–17. doi: 10.1006/jmbi.2000.4042 . 10964570 10.1006/jmbi.2000.4042

[36] Gouet P, Robert X, Courcelle E. ESPript/ENDscript: Extracting and rendering sequence and 3D information from atomic structures of proteins. Nucleic Acids Res. 2003;31(13):3320–3. Epub 2003/06/26. ; PubMed Central PMCID: PMC168963. 12824317 10.1093/nar/gkg556 PMC168963

[37] Dundas J, Ouyang Z, Tseng J, Binkowski A, Turpaz Y, Liang J. CASTp: computed atlas of surface topography of proteins with structural and topographical mapping of functionally annotated residues. Nucleic Acids Res. 2006;34(Web Server issue):W116–8. doi: 10.1093/nar/gkl282 ; PubMed Central PMCID: PMCPMC1538779. 16844972 10.1093/nar/gkl282 PMC1538779

[38] Arnold K, Bordoli L, Kopp J, Schwede T. The SWISS-MODEL workspace: a web-based environment for protein structure homology modelling. Bioinformatics. 2006;22(2):195–201. Epub 2005/11/23. doi: 10.1093/bioinformatics/bti770 . 16301204 10.1093/bioinformatics/bti770

[39] de Beer TA, Berka K, Thornton JM, Laskowski RA. PDBsum additions. Nucleic Acids Res. 2014;42(Database issue):D292–6. doi: 10.1093/nar/gkt940 ; PubMed Central PMCID: PMCPMC3965036. 24153109 10.1093/nar/gkt940 PMC3965036

