# Supplementary figures and images for: The structure of FIV reverse transcriptase and its implications for non-nucleoside inhibitor resistance
Source: PLoS Pathog. 2018 Jan 24;14(1):e1006849. doi: 10.1371/journal.ppat.1006849 (PMC5798851; doi:10.1371/journal.ppat.1006849)

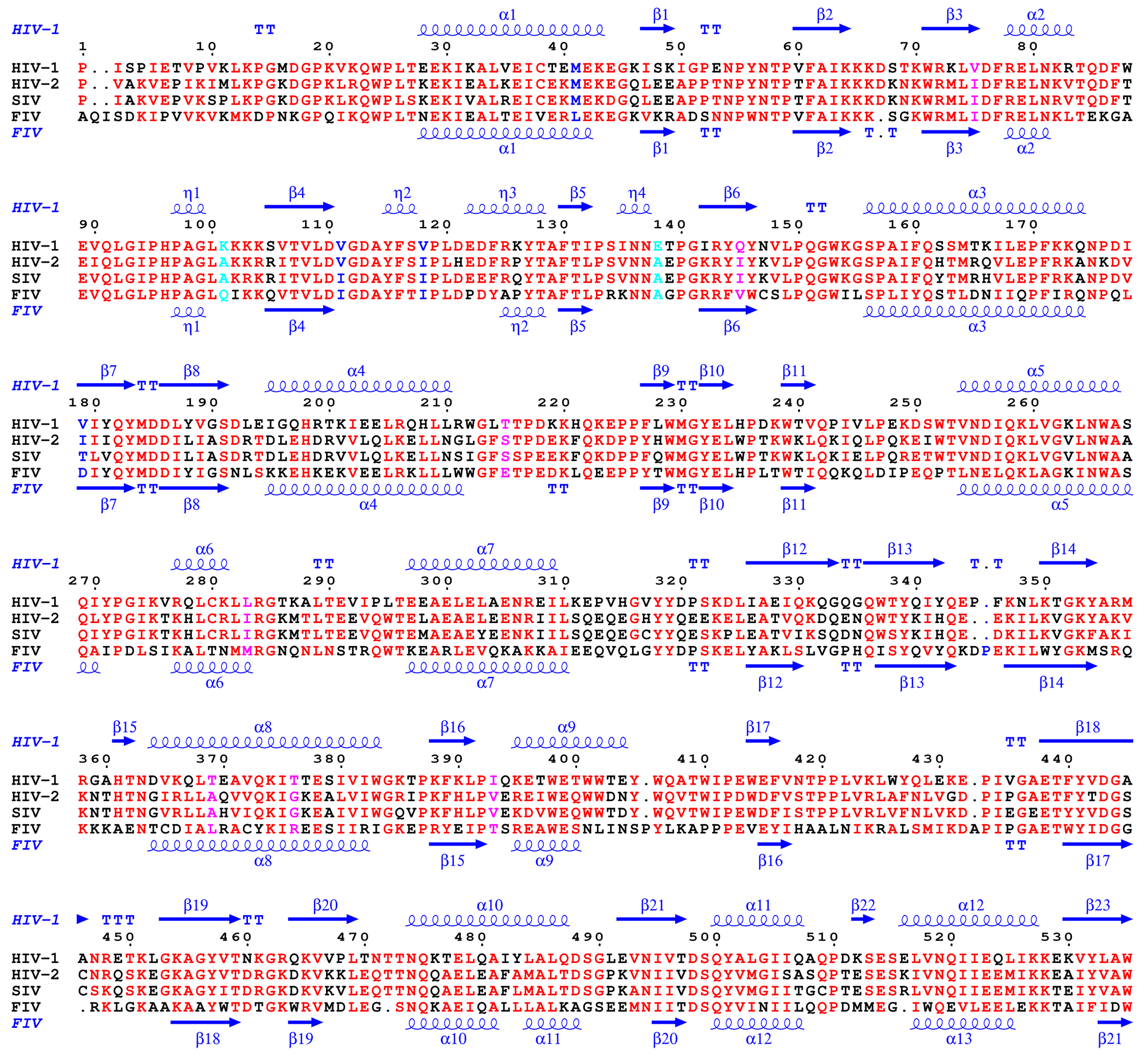

Supplement: S1 Fig — Sequences of RT from HIV-1, HIV-2, SIV and FIV are compared. Top numbering and secondary structure are shown for HIV-1 (PDB code: 5HBM) and are shown for FIV RT at the bottom. Identical residues are shown in bold red font and conserved equivalents in red font. NNRTI-resistance mutations known in HIV-1 and intrinsic in FIV are in blue. FIV substitutions in known HIV-1 NNRTI-resistance positions are in pink except for two well-investigated mutations K101 and E138 in cyan. (TIF) [file ppat.1006849.s001.tif]

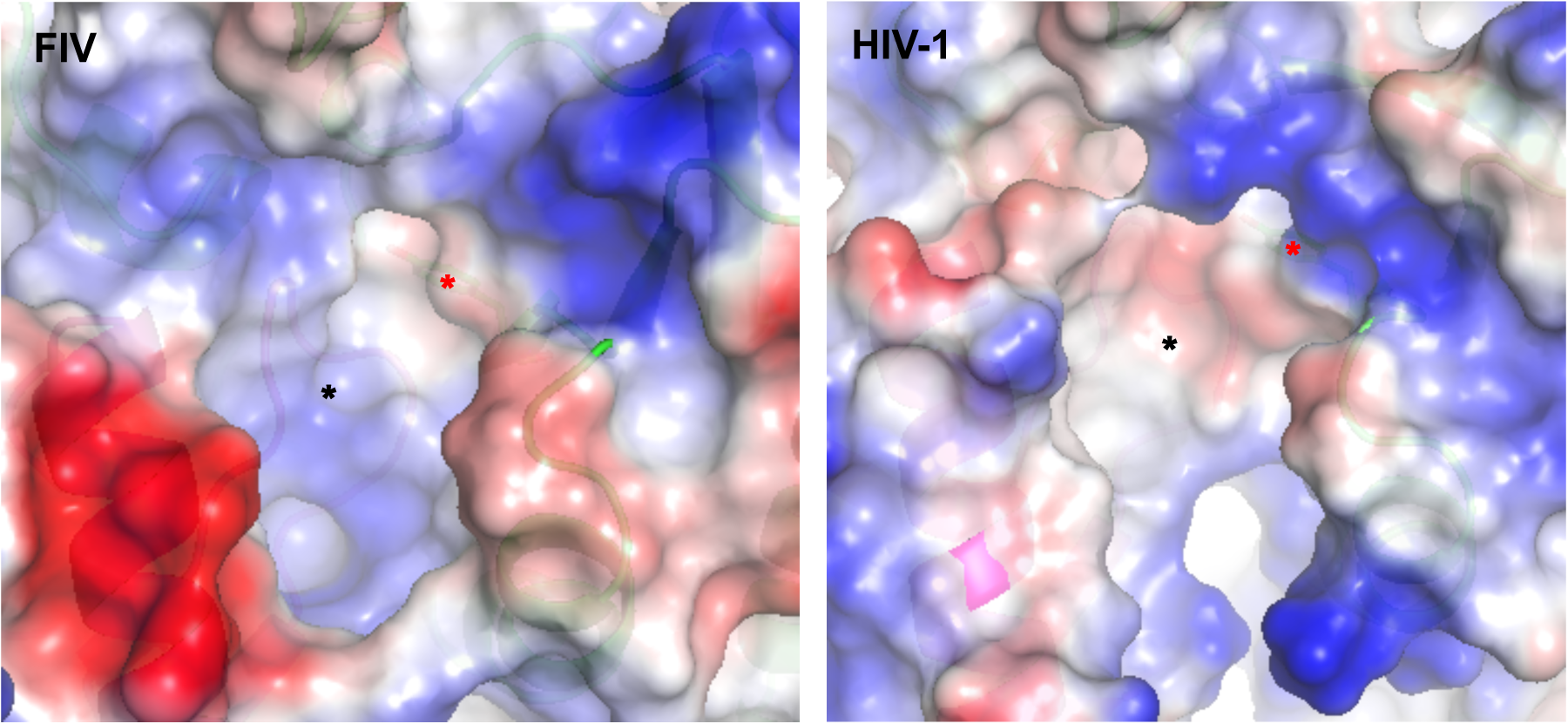

Supplement: S2 Fig — Electrostatic surfaces of RT from FIV (left) and HIV-1 (right, PDB code: 1DLO) are displayed over cartoon representation. Indicated are Y181 (red asterisk) and p51 loop (black asterisk), which is positively charged (blue) in fRT and negatively charged (red) in HIV-1 RT. (TIF) [file ppat.1006849.s002.tif]

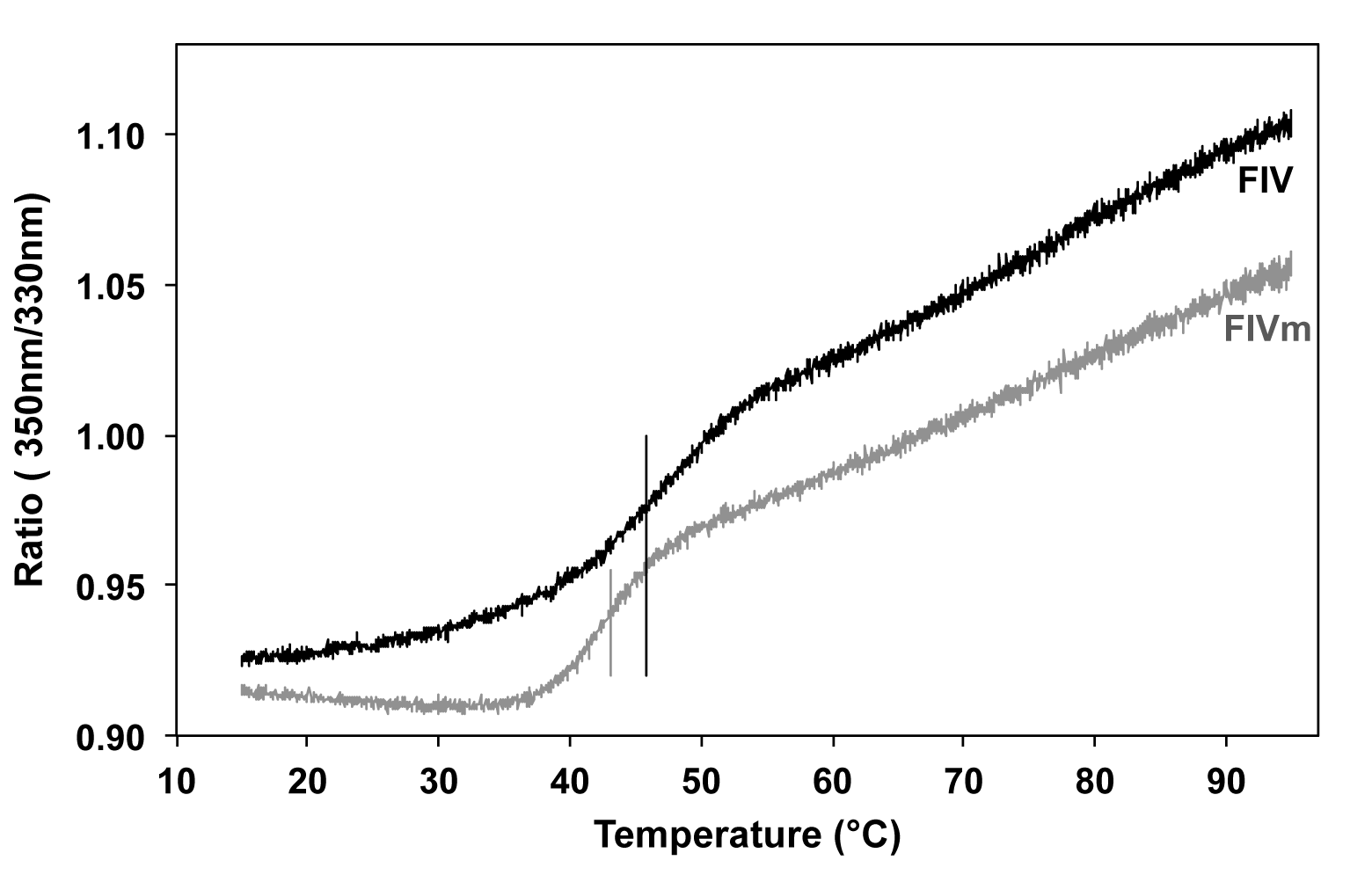

Supplement: S3 Fig — A ratiometric measurement of the fluorescent signal (Ratio) is plotted against increasing temperature for wild type FIV RT (FIV, black line) and the p51 mutant (FIVm, gray line). The melting temperatures (Tm) of wild type RT (45.8 ± 0.7°C) and mutant (43.1 ± 0.1°C) are mean values (with standard deviations) of two repeats (each with triplicate measures) and are presented with black and gray vertical lines, respectively. The difference in Tm (2.7°C) is statistically significant (P-value = 0.0004, one-way ANOVA). For clarity, the Y-axis scale of FIVm is shifted downwards by 0.03 ratio points. (TIF) [file ppat.1006849.s003.tif]

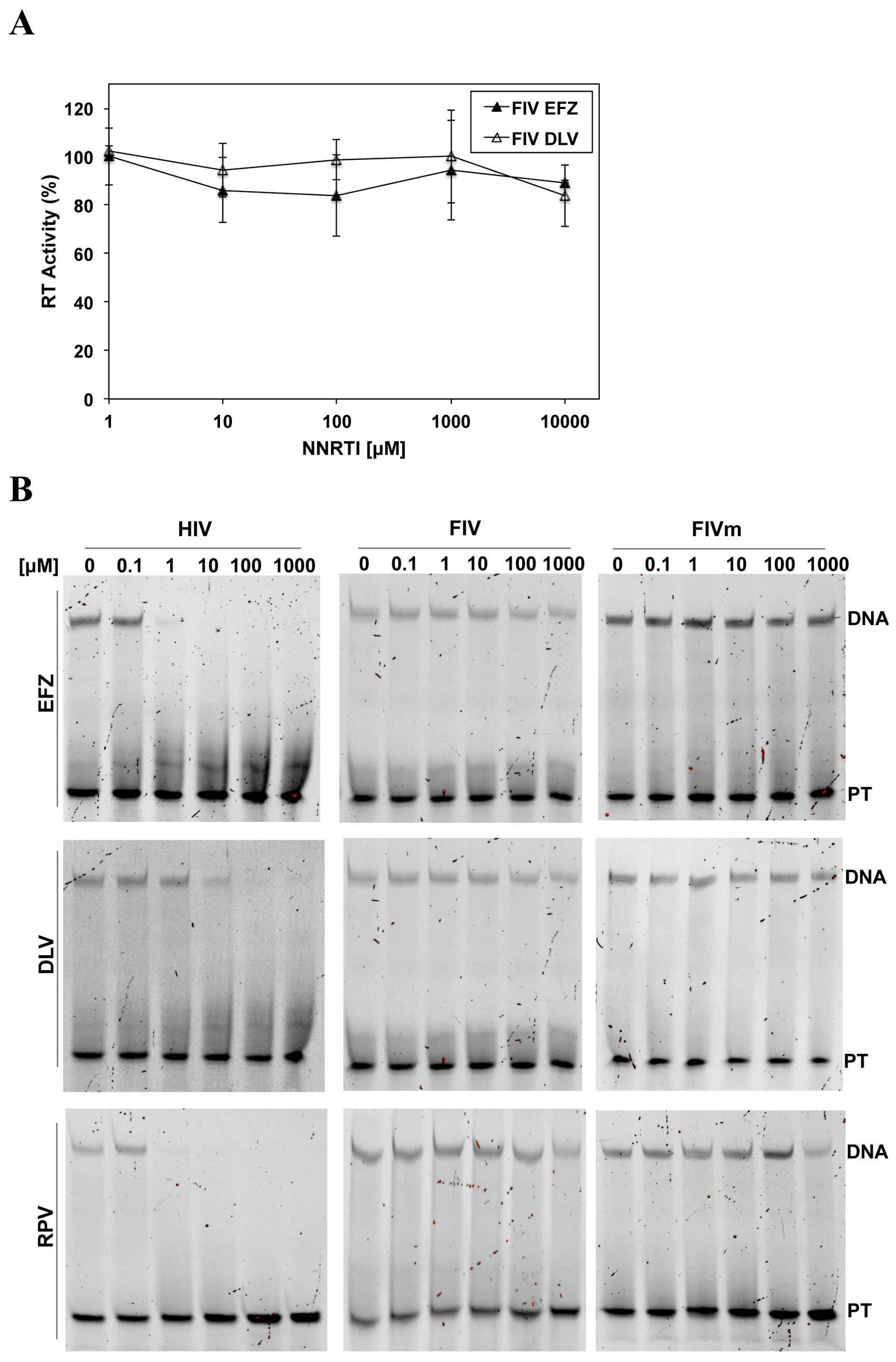

Supplement: S4 Fig — (A) DNA polymerization activity of wild type FIV RT as affected by EFZ (black marks) and DLV (clear marks). (B) Representative gels used in the quantification of NNRTI effect on activity. Gels show the resolution of the fluorescent-primer/template substrate (PT) and the polymerization product (DNA), which is fluorescently labeled only if the fluorescent-primer was elongated by RT activity. (TIF) [file ppat.1006849.s004.tif]

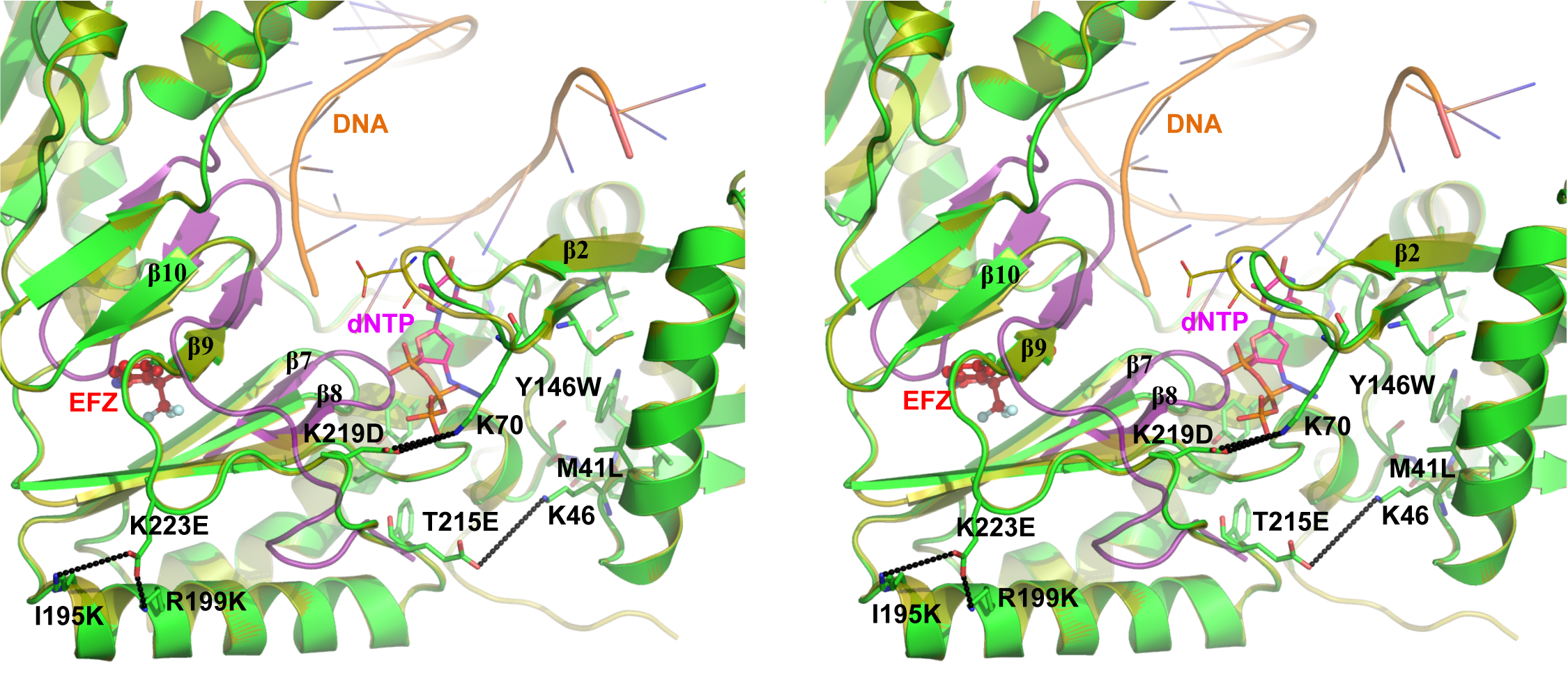

Supplement: S5 Fig — Walleye stereo view of FIV RT (green cartoon) after being modeled bound to DNA (orange) and dNTP (magenta sticks) using HIV-1 RT complex structure (dark yellow, PDB code: 3V4I). EFZ (red stick-spheres) and dislocated primer-grip (β9/10) and catalytic-loop (β7/8) (purple cartoon) are modeled using was modeled using 1FK9. Potential salt bridges are indicated with black dashed lines. (TIF) [file ppat.1006849.s005.tif]
